# ScaffViz: visualizing metagenome assemblies

**DOI:** 10.1186/gb-2011-12-s1-p8

**Published:** 2011-09-19

**Authors:** Sergey Koren, Todd Treangen, Mihai Pop

**Affiliations:** 1 Department of Computer Science, University of Maryland, College Park, MD 20742, USA; 2 Center for Bioinformatics and Computational Biology, University of Maryland, College Park, MD 20742, USA; 3 The McKusick-Nathans Institute for Genetic Medicine, The Johns Hopkins University School of Medicine, Baltimore, MD 21205, USA

## Background

Metagenomics has allowed the study of a wide range of microbial communities, from those within the sea [1,2] to those of the human body [3]. Increasingly, *de novo* assembly is the first step in the analysis of these metagenomic samples. As the targets have increased in complexity, computational tools have started to emerge [4,5] to address the challenges presented by the assembly of these datasets. Although the targets and analyses have become more complex, the means of presenting the results has remained the same: a multi-FASTA text file. This presentation hides the variation that is present in the sampled biological community. The ability to navigate and view the complexity of a genomic sample may help drive novel biological insights. Here, we present a graphical visualization tool that allows the visual inspection of genome assembly graphs and the characterization of the genomic variation that is present in these graphs (that is, the differences between two or more related haplotypes commonly found in metagenomes or higher eukaryotes).

## Methods

Our software, ScaffViz [6], is open source and was developed as a plug-in for the Cytoscape graph viewer package [7,8]. Our assembly view represents assembly metadata within node/edge attributes. For example, node height corresponds to coverage (the amount of oversampling of a sequence), and node width is proportional to the length of the sequence. We support assemblies from Celera Assembler [9], Newbler [10], Bambus 2 and MetAMOS. The creation and initialization of Cytoscape objects is abstracted to allow a developer to easily add new assembly result formats without knowledge of Cytoscape’s API. We developed a layout algorithm based on information from the assembler on node position, orientation and length. ScaffViz allows users to show (or hide) an arbitrary subset of nodes. The viewer can also output genome sequence that corresponds to any subset of the graph, including all alternative sequences present in all selected subpaths. We believe that this representation may prove to be instrumental in finding and characterizing structural variants such as alternative genes, alternative regulatory units or mobile genomic elements.

## Results

We evaluated the performance of ScaffViz on seven datasets of varying size and complexity. We report that the run time is approximately linear with respect to the number of elements in the graph (nodes + edges). The memory scales linearly with respect to the number of nodes. Extrapolating from these factors, a graph of 250,000 contigs can be opened in approximately 2 minutes using approximately 2.5 GB of memory. ScaffViz is scalable to large graphs and can be run on a laptop.

## Conclusions

We have developed a novel open-source assembly graph viewer, ScaffViz, as a plug-in for Cytoscape. ScaffViz supports the output of several popular assembly programs and is scalable to large metagenomic assemblies on a laptop.
